# Evaluating the Elemental Composition, Transformation Behavior, Crystalline Structure, and Mechanical Properties of Three 0.016-Inch by 0.022-Inch Nickel-Titanium Archwires: An In Vitro Study

**DOI:** 10.7759/cureus.27206

**Published:** 2022-07-24

**Authors:** Odayy Al-Horini, Mohammad Y Hajeer, Feras Baba

**Affiliations:** 1 Department of Orthodontics, University of Aleppo Faculty of Dentistry, Aleppo, SYR; 2 Department of Orthodontics, University of Damascus Faculty of Dentistry, Damascus, SYR

**Keywords:** nickel-titanium wires, mechanical properties, transformation behavior, elemental composition, archwires, temperature of end transition, crystalline structure, thermal nickel-titanium wires, unloading force

## Abstract

Background

Nickel-titanium (NiTi) archwires are considered the most attractive wires during the first stage of orthodontic treatment because of their unique properties throughout several generations. This study aimed to evaluate three different NiTi wires in terms of their elemental composition, transformation behavior, crystalline structure, and mechanical properties.

Materials and methods

The study used three different groups of NiTi archwires with dimensions of 0.016 x 0.022-inch (American Orthodontics®, Sheboygan, WI, USA). The first group included six superelastic NiTi archwires (NT3-SE®), with normal force and a stable structure that was not affected by temperature changes. The second group included six heat-activated NiTi archwires activated at 25°C (Thermal Ti-D®), with moderate force and a sensitive structure to thermal changes, especially at room temperature. The third group included six heat-activated NiTi archwires activated at 35°C (Thermal Ti-Lite®), with light force and a sensitive structure to thermal changes, especially at body temperature. X-ray fluorescence (XRF) was performed to determine wire element composition, whereas differential scanning calorimetry (DSC) was performed to determine the austenite finish temperature (A_f_). The X-ray diffraction (XRD) analysis was used to identify the crystalline structure at room temperature, and a three-point bending test was carried out under constant temperature (37°C) with respect to the instructions of ISO15841/DIS to evaluate the mechanical properties of these wires.

Results

The XRF analysis revealed that the superelastic NiTi archwires (NT3-SE) were composed of NiTi and chrome, whereas the heat-activated wires (Thermal Ti-D and Thermal Ti-Lite) were composed of NiTi and copper. The DSC showed the A_f _was at +16.84°C for the superelastic type (NT3-SE), +23°C for the heat-activated at 25°C (Thermal Ti-D), and +33.99°C for the heat-activated at 35°C (Thermal Ti-Lite). The XRD analysis identified the crystalline structure at room temperature for the superelastic type (NT3-SE) as austenite, while for thermal types (Thermal Ti-D and Thermal Ti-Lite) were a compound structure of austenite and martensite phase. Finally, the bending test showed that the highest forces were delivered from the superelastic type (NT3-SE), followed by heat-activated at 25°C (Thermal Ti-D), while the lowest forces were delivered from heat-activated at 35°C (Thermal Ti-Lite). There was no significant difference between the superelastic type (NT3-SE) and thermally activated type at 25°C (Thermal Ti-D), while there was a significant difference between the two previous types and the thermally activated type at 35°C (Thermal Ti-Lite) for all studied unloading points.

Conclusions

The thermal types of archwires (Thermal Ti-D and Thermal Ti-Lite) had lower unloading values in comparison with the superelastic type (NT3-SE). The elemental composition was different between the superelastic wires and the thermal ones. The superelastic wires were also different from the other two types in terms of crystalline structure. The three types of archwires had an activation degree located in the range of oral cavity variations.

## Introduction

In the early 1970s, Andreasen and Hilleman suggested nickel-titanium (NiTi) alloys for manufacturing orthodontic archwires [1]. Since then, several generations and classifications of NiTi archwires have appeared. Kusy [2] divided NiTi wires into three major groups: the conventional NiTi group, which contains archwires with a stable martensitic phase, a high degree of the transformation temperature range (TTR), and are considered non-elastic Nitinol archwires. The second group (pseudo-elastic NiTi group) contains archwires that have an austenitic active phase at room temperature, some of shape memory effect the phase transition process under the effect of stress-induced martensite (SIM). The third group, the thermoelastic NiTi group, contains archwires with a martensitic active phase, shape memory effect, TTR located in the range of oral cavity temperature, and both the transition and the reverse transition processes are under the effect of thermal changes [2-4]. NiTi archwires are considered the most attractive wires to orthodontists because of their high superelastic properties, which allowed having a wide range of activation and pending, in addition to their production of low force level [2-4].

The orthodontic archwires used in the initial stage of treatment must produce a continuous light force, measured around 50 grams, which is enough to produce tipping movement in this stage of treatment [3]. Superelastic NiTi archwires usually are considered the best choice to achieve this aim [5], which later can be replaced with other types of archwires such as beta-titanium or stainless steel. Both superelastic and thermal NiTi archwires are useful in cases that need wide deformation of the wire to engage the brackets as in severe crowding [2-4].

In light of some in vitro studies evaluating NiTi wires, [1,5-8] many authors have concluded that several factors play an essential role in the behavior of NiTi orthodontic wires with an emphasis on the wire generation. Andreasen and Hilleman [1] evaluated a high force level from the first generation of NiTi wires at a 3-mm deflection, whereas Burstone et al. [5] found that superelastic NiTi wires (second generation) provided spring back that is 1.6 times that of the Nitinol of the previous generation. Another important aspect is the elements that compose the orthodontic wires. Nespoli et al. [6] noticed the microstructure's effect on each archwire's mechanical response, especially for copper NiTi wires, which showed lower thermal and mechanical hysteresis and thermal sensitivity effect. Iijima et al. [7] showed that change in the surrounding temperature resulted in producing temporary change on superelastic wires. In contrast, this change was a little bit more on the thermal NiTi wires. Kawashima et al. [9] attributed the change in force level between NiTi wires to the difference at austenite finish temperature and the amount of applied deformation in the same field. Parvizi and Rock [10] found an increase in unloading forces relative to an increase in deflection.

The mechanical behavior of NiTi wires through its several generations depends on the physical situation and the dramatic changes that happened to the crystalline structure [2-5,7,10]. Higa et al. in their study focused only on the small dimensions of NiTi wires [11], while Lombardo et al. studied the mechanical behavior of NiTi wires without searching the physical situations [12]. On the hand, Iijima et al. studied only the physical structure of samples from NiTi wires [13] without trying to find the relationship between both mechanical status and physical status.

This study aimed to determine the differences in mechanical behavior between three commercial NiTi wires; one of them was the superelastic wire, whereas the other two types were thermal heat-activated wires at two different temperature degrees (25°C and 35°C). In addition, this study aimed to detect the elements composing these wires, the phase transformation, and determine the transitions temperature range, by using three-point bending test, X-ray fluorescence (XRF), X-ray diffraction (XRD), and differential scanning calorimetry (DSC). The null hypothesis was that there were no differences between the three types of NiTi wires in terms of their mechanical properties, elemental composition, transformation behavior, and crystalline structure.

## Materials and methods

Study design and settings

This study was a single-center, single-blind randomized controlled in vitro study for three different types of NiTi archwires. This study was conducted at the Department of Orthodontics, Aleppo Dental School, Aleppo University, Syria, in collaboration with the Faculty of Mechanical Engineering at Aleppo University, Syria, and the Higher Institute for Applied Sciences and Technology (HIAST).

Sample size calculation

The sample size was calculated using Minitab® Version 17(Minitab Inc., State College, Pennsylvania, PA, USA) based on the study by Bavikati et al. [14], which evaluated the superelastic NiTi wires. The standard deviation in this study was 19.86. The mean difference of the tensile forces between different brands of superelastic wires was -8.31 Mpa. At a significance level of 5% and a power of 90%, six archwires were found required for each group.

The experimental groups 

The first group included superplastic NiTi wires, which are not affected by temperature degree change and stable, moderate force levels according to the claim of the manufacturer (NT3-SE®, American Orthodontics, Sheboygan, WI, USA). The second group included thermal NiTi wires activated over 25°C (room temperature) and with moderate force levels according to the claim of the manufacturer (Thermal Ti-D®, American Orthodontics). The third group included thermal NiTi wires activated over 35°C (body temperature) and with light force levels according to the claim of the manufacturer (Thermal Ti-Lite®, American Orthodontics). All wires in the three groups had a dimension of 0.016 x 0.022 inch; the length of tested samples was 30 mm, cut from the posterior section of the arch by a heavy straight cutter.

Outcome measures

Elemental Composition 

The elements that composed the archwires were assessed using the XRF technique (XMF-104®, Unisantis Holding Company Limited, Georgsmarienhutte, Germany), which is a fast and useful method to define elements without damaging tested samples [6,7,11,12].

Load/Deflection Curves

**Figure 1:**
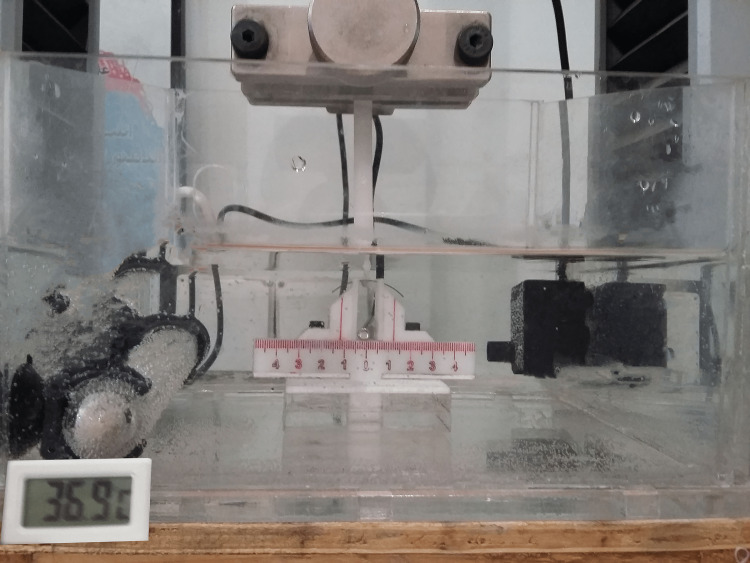
The water bath used to conduct the three-point bending test with a constant temperature of about 37 centigrade degrees.

The three-point bending test was performed using a universal testing machine (Testometric 350M, Instron, England) with a modified load cell 100N and a water bath made from Plexiglas to stimulate a stable temperature at 37°C [12]. The length of the tested wires was 30 mm, while the length of spam was 10 mm. Each wire was deflected to 3.1 mm by crosshead with 1 mm/minute speed rate and pointed contact on the center of the tested wire (Figure 1). The deflection was done in the vertical direction (occlusal-gingival), and the setting of this experiment was done in accordance with the guidelines of ISO/DIS 15841 [15]. The resulted load/deflection curves were analyzed using Solid Works (SolidWorks-v2012, Dassault systems, Concord, MA, USA) and Microsoft Excel (Microsoft Excel®2013, Microsoft Corporation, Redmond, WI, USA). The unloading forces were evaluated at four points: 0.5, 1, 2, and 3 mm. These points expressed the deflection of wire and the magnitude of forces that the tooth would receive during the start (i.e, 3 mm) and the end (i.e., 0.5 mm) of the unloading stage; the forces were compared between the three different groups.

The Crystalline Structure

The crystalline structure of the atoms and the diffraction pattern was evaluated using the X-ray Diffractometer® (XRD; Bruker AXS, Karlsruhe, Germany) with a Cu-Ka radiation at 40 kV and 300 mA. The XRD test was performed on one sample with a dimension of 1 cm2 for each type of wire; the wires sat side by side to reach that size, while the temperature was set at 25°C (room temperature) and increased gradually to 90°C.

Thermal Behavior

Finally, the transformation temperature for each wire was examined by using DSC (DSC131, Setaram, Caluire, France), which is one of the thermal analysis techniques used in studying the thermal transition of different materials [8,9,10,16,17]. The temperature during the assessment ranged from -100°C to +100°C, and the scanning was performed at a rate of 10 c/minute.

Statistical analysis

The SPSS program V20 (IBM, Armonk, NY, USA) was used for statistical analysis. Kolmogorov-Smirnov tests were to evaluate the normality of the distributions. One-way analysis of variance (ANOVA) tests were used for the analysis of variance, whereas post-hoc Sidak's tests were used to detect significant differences between each pair of groups with a level of significance set at 5%.

The error of the method

Three different wires from the tested sample were chosen randomly with the concern to be one wire from each different group to repeat the load/deflection test. Then, the intra-class correlation coefficients (ICCs) were calculated to check the reliability of the method. The result showed high reliability since all the ICCs were greater than 0.984.

## Results

Elemental composition

The XRF analysis revealed that the superelastic NiTi archwires (NT3-SE) were composed of NiTi and chrome, whereas the heat-activated wires (Thermal Ti-D and Thermal Ti-Lite) were composed of NiTi and copper (Table 1).

**Table 1:** Elemental composition of the archwires used in the current study Ni, nickel; Ti, titanium; Cr, chromium; Cu, copper

Commercial name of archwires	Type	Elements composition
NT3-SE	Superelastic	Ni-Ti-Cr (50%-48%-2%)
Thermal Ti-D	Heat-activated at 25°C	Ni-Ti-Cu (49%-48%-3%)
Thermal Ti-Lite	Heat-activated at 35°C	Ni-Ti-Cu (49%-47%-4%)

Load/Deflection Curves

**Figure 2:**
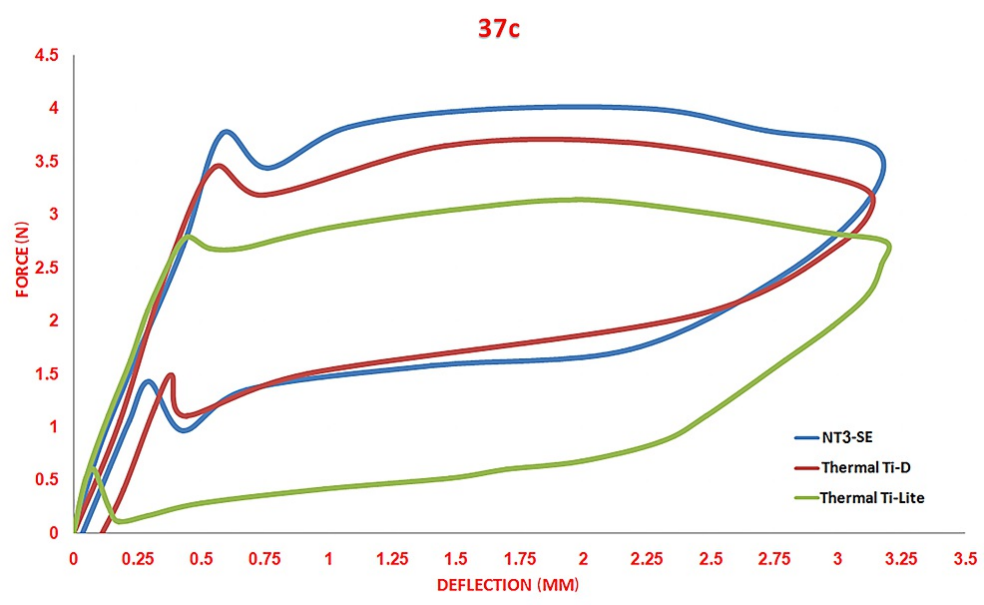
Load/deflection curves of the three different nickel-titanium archwires at a stable temperature of 37°C.

All the tested wires showed a classic load/deflection curve of NiTi alloys, which is distinguished by the flatting of the plateau during the loading and unloading stage (Figure 2). Thermal Ti-Lite type delivered the lowest forces in comparison with other wires, while NT3-SE type delivered the highest force level (Table 2).

**Table 2:** Descriptive statistics of the unloading forces for the tested three types of archwires. SD, standard deviation; Max, maximum; Min, minimum

Unloading value	Types of archwires											
	NT3-SE				Thermal Ti-D				Thermal Ti-Lite			
	Mean	SD	Max	Min	Mean	SD	Max	Min	Mean	SD	Max	Min
0.5 mm	1.09	0.19	1.35	0.85	0.89	0.15	1.10	0.70	0.38	0.20	0.70	0.15
1 mm	1.40	0.18	1.65	1.15	1.25	0.13	1.45	1.10	0.52	0.09	0.65	0.40
2 mm	1.60	0.18	1.95	1.40	1.44	0.11	1.60	1.30	0.76	0.10	0.90	0.60
3 mm	2.65	0.11	2.80	2.50	2.50	0.11	2.65	2.35	2.18	0.15	2.35	1.90

For the statistical evaluation, the difference among primary outcomes (the unloading forces at the following four deflections: 0.5, 1, 2, 3 mm) for the three types of wires was tested by comparing the mean values with ANOVA. Sidak's post-hoc tests were used to detect significant differences for pairwise comparisons. There was no significant difference between the superelastic type (NT3-SE) and thermally activated type at 25°C (Thermal Ti-D; p<0.05), while there was a significant difference between the two previous types and the thermally activated type at 35°C (Thermal Ti-Lite) for all studied unloading points (p<0.01). NT3-SE type showed the highest unloading values followed by Thermal Ti-D, whereas Thermal Ti-Lite showed the lowest values (Table 3).

**Table 3:** Post-hoc tests for pairwise comparisons between the three types of archwires regarding the three-point bending tests. NS, not significant

Unloading value	Comparing wires	Significance
0.5 mm	NT3-SE vs. Thermal Ti-D	NS
	NT3-SE vs. Thermal Ti-Lite	<0.001
	Thermal Ti-D vs. Thermal Ti-Lite	0.014
1 mm	NT3-SE vs. Thermal Ti-D	NS
	NT3-SE vs. Thermal Ti-Lite	<0.001
	Thermal Ti-D vs. Thermal Ti-Lite	<0.001
2 mm	NT3-SE vs. Thermal Ti-D	NS
	NT3-SE vs. Thermal Ti-Lite	<0.001
	Thermal Ti-D vs. Thermal Ti-Lite	<0.001
3 mm	NT3-SE vs. Thermal Ti-D	NS
	NT3-SE vs. Thermal Ti-Lite	<0.001
	Thermal Ti-D vs. Thermal Ti-Lite	0.032

Crystalline structure and thermal behavior

**Figure 3:**
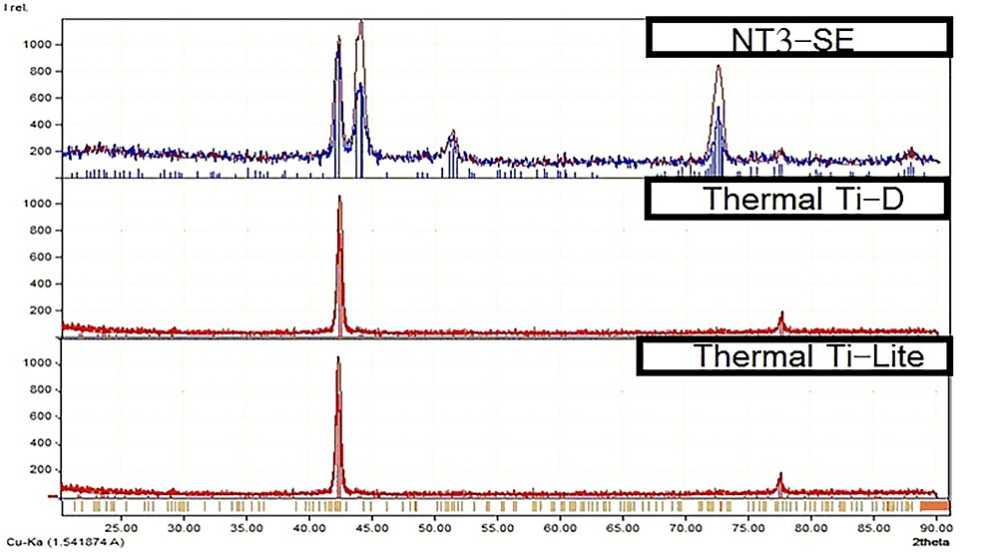
The X-ray Diffractometer® (XRD) analysis for each type of the evaluated archwires. (A) NT3-SE. (B) Thermal Ti-D. (C) Thermal Ti-Lite.

Both thermal wires (Thermal Ti-D and Thermal Ti-Lite) showed two peaks (at 002°F and 110°F) during the XRD test, while the superelastic type (NT3-SE) showed only one specific peak (at 110°F; Figure 3).

**Figure 4:**
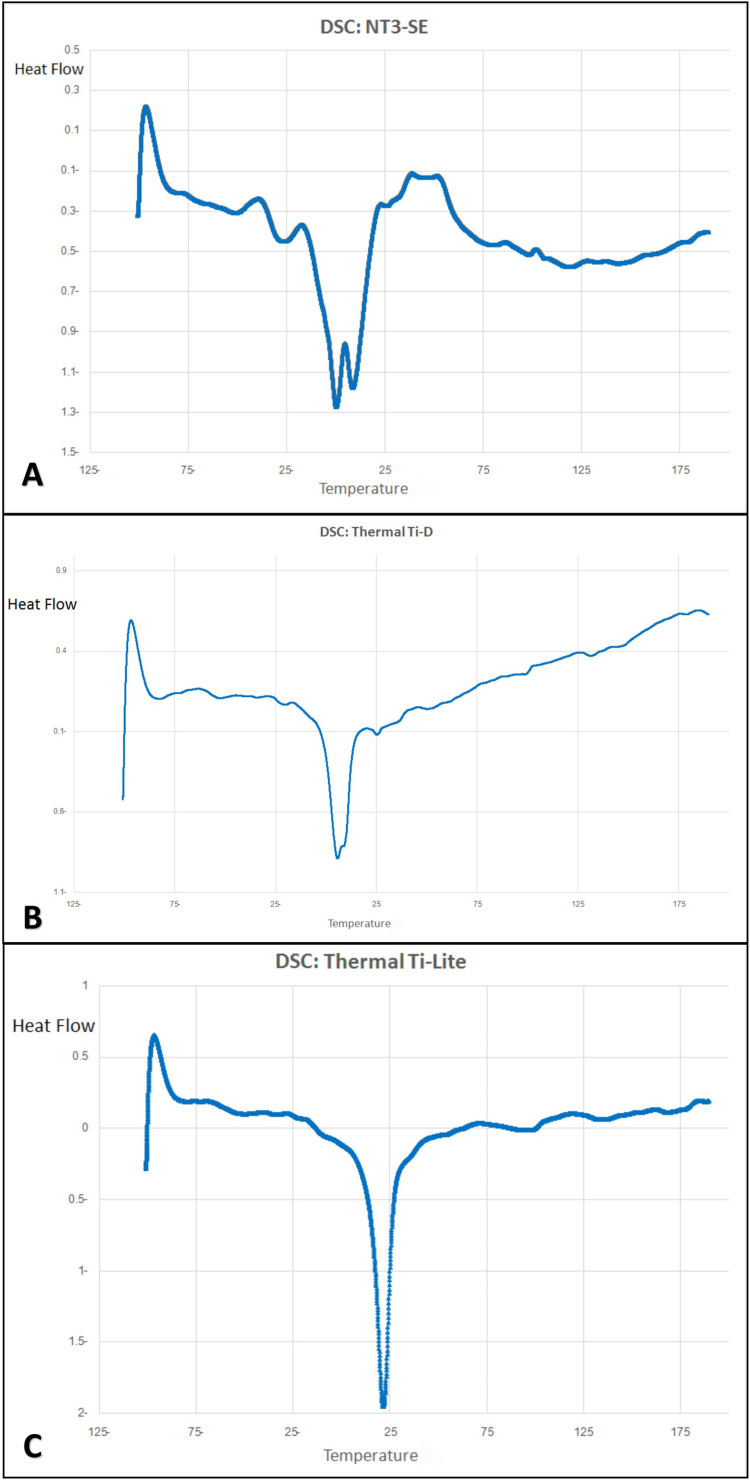
DSC curves of the three different nickel-titanium wires. DSC, differential scanning calorimetry

On the other hand, the thermal NiTi wires (Thermal Ti-D and Thermal Ti-Lite) showed one peak during the heating tour between -100°C and +100°C using the DSC test (Table 4; Figure 4). Thermal Ti-Lite showed the highest temperature degrees at both the austenite-start and austenite-finish temperatures (A_s_ = +15.02 and A_f_ = +33.99°C), while Thermal Ti-D showed the lowest temperature degrees for the austenite-start, but a temperature for the A_f _near to the room temperature (A_s_ = -1.47 and A_f_ = +23°C). Only the superelastic type (NT3-SE) showed two peaks during the heating tour, with a moderate austenite-start temperature degree lying between the two thermal types, whereas the A_f_ temperature was the lowest and below the room temperature (A_s_ = +8.73 and A_f_ = +16.84°C).

**Table 4:** Results obtained from the differential scanning calorimetry analysis.

Archwires	R-phase (in °C)		Austenite phase (in °C)	
	R-start	R-finish	A-start	A-finish
NT3-SE	-8.79	+0.83	+0.38	+16.84
Thermal Ti-D	-	-	-1.47	+23
Thermal Ti-Lite	-	-	+15.02	+33,99

## Discussion

The unique property of NiTi archwires among the different generations used in orthodontics is the ability to apply light, stable, and continuous forces with a wide range of activation [3-5,7,9,18]. This property is due to the phase transition at the crystalline structure [4,7]. It happens either under the effect of externally applied forces as in super elastic NiTi arches generation or temperature changes as in thermal NiTi heat-activated branches [2,7,9]. During orthodontic treatment, engaging NiTi arch into brackets incites the start phase transition. The transition occurs between austenite and the martensite phase and is called stress-induced martensite (SIM) [2,3,7]. It happens in most NiTi generations of archwires, while the reverse transition occurs at the unloading stage and corresponds with the biological reaction of the tooth during orthodontic movement, affected mainly by wires generations [2,3,7,9]. On the other hand, thermal wires showed lighter forces than the superelastic category and this could be attributed to the temperature effect on the crystalline structure and the chemical composition [6,7,9,10].

In the current study, three different wire types with the same dimension (0.016 x 0.022 inch) and from the same manufacturer were used for the bending test. The bending test is the most mechanical test that approaches what happens during wire bending and teeth reaction against this bend when simulating orthodontic movements [3,5,7,9,10]. In general, bending tests show two types of results: loading values that correspond to the forces applied to engage the wires through brackets and unloading values that express forces delivered to teeth from archwires. Both of these loading and unloading curves show a flattening after a proximal deflection in most of the NiTi wire generations [2,3,9]. We compared the unloading values between wires at unloading points (0.5-1-2-3 mm), which were defined from the instructions of ISO\DIS 15814 [15]. As we expected, the thermal type with heat activation at 35°C (Thermal Ti-Lite) showed the lowest unloading value at the four tested points, while the superelastic type (NT3-SE) showed the highest values. The thermal types activated at 25°C had a moderate value compared to the other types. Flattening in both of the loading and unloading curves appeared, which means that the three wire types had a phase transition during loading and a reverse phase transition during unloading.

Usually, NiTi alloys show thermal sensitivity among different generations [2,8]; this sensitivity appears as increasing generated forces due to changes in the crystalline structure that clearly appeared in the thermal branch when it was tested over its A_f_ temperature [2,7,9]. However, when unloading forces were compared between the superelastic type (NT3-SE) and the heat-activated at 25°C type (Thermal Ti-D), no significant differences were detected, with forces delivered from the superelastic type being higher than the thermal type.

Actually, the temperature test of 37°C was greater than the A_f_ temperature for both types (+16.84; NT3-SE, 23°c; Thermal Ti-D); therefore, the crystalline structure at 37°C for both types was the same. Specifically, over 25°C, both types have an austenite structure, and that explains the convergent in force levels. On the other hand, we found a primary significant difference between previous wires and heat-activated type at 35°C (Thermal Ti-lite) in all unloading points. Maybe it is related to the difference in A_f_ temperature between wires, whereas this type had a higher A_f_ temperature (33,99°c; Thermal Ti-lite) than the other types. This is in agreement with many authors' conclusions that wires with high A_f_ temperature show lower stiffness and forces in a confrontation of wires with lower A_f _temperature [2,7,9,10,18].

X-ray diffraction 

NiTi alloys could show two different crystalline structures, austenite and martensite, Each structure shows different mechanical properties and produces different force levels. The change between these two structures can be obtained under the effect of stress or temperature changes [3-5,7]. XRD is one of the best methods that can be used to identify the crystalline structure and phase transition, as this method depends on the diffraction of X-Ray waves between atoms that shape crystallizes and drawing curves that express the spaces between these atoms [7, 9,18,19]. Analyzing those curves can help identify the current phase, and each curve includes peaks, which relate to the spaces between atoms and its relations in 3D structures.

In XRD patterns of NiTi alloys, both peaks 110 and 002 are the most important in the identification of the crystalline phases; 110 peak is related to the appearance of the austenite phase, while the 002 peak is related to the martensite phase [7,18,20]. In our study, we had performed an XRD test for one sample of each type of wire, at room temperature, without application of any tensile or compression (as received), and the resulted XRD patterns were analyzed using Match v3.1 soft ware (MATCH, phase identification from powder diffraction, Crystal Impact, Bonn, Germany).

Both types (NT3-SE and Thermal Ti-D) showed a single peak (110), the specific peak of the austenite phase; the appearance of that peak assured us that the phase of those two types at room temperature (25°C) without applying any compression force (as received) is full austenite, and that is in agreement with the results from DSC, which show an austenite finish temperature (+16.84°C) for the superelastic (NT3-SE) type and (+23°C) for the thermal type (Thermal Ti-D). Both austenite finish (A_f_) degrees were lower than the test degree, and thus the phase will be austenite under the effect of temperature for those types. On the other hand, thermal wires (Thermal Ti-Lite) showed the appearance of two peaks: the peak 002, which is related to the martensite phase, and the peak 110, which is related to the austenite phase. That means that this type did not exhibit a full transition into the austenite phase at room temperature (25°C), and thus it showed both crystalline structures (austenite-martensite), and that agrees with the DSC result, whereas A_f_ for (Thermal Ti-D) was +33.99°C, i.e., higher than the room temperature. Therefore, this type did not have a complete transition to the austenite phase under the effect of temperature, and had a martensite structure with some austenite structure.

DSC analysis

The most effective factor in the mechanical behavior of thermal NiTi wires is A_f_ (austenite finish) [8-9]. At this temperature usually NiTi alloys had full phase transitions from the martensite phase to the austenite phase under change and rise of temperature over this degree without the interposition of R-phase [8].

By using DSC, we determined A_f_ for three commercial wires and detected peaks, which refer to transition between phases. The values of A_f_ for thermal wires were 23°C for Thermal Ti-D (Ni-Ti-Cu; 49-48-3) - heat activated at 25°C as manufactory allegation - and 33.94°C for Thermal Ti-Lite (Ni-Ti-Cu; 49-47-4) - heat activated at 35°C as manufactory allegation. For two thermal wires, one peak had been detected by analyzing DSC curve; this peak related to the transition from the martensitic phase to the austenitic phase without interposition in the R-phase. Thermal NiTi wires usually contain in their composition an additional metallic element such as copper; this addition to alloys aims to raise the austenite finish temperature [2,6]. Both thermal wires used in the study contained different amounts of copper, and this element plays an important role in different A_f_ temperatures. The difference in the A_f_ between Thermal Ti-D and Thermal Ti-Lite may be related to the amount of copper and manufacturing conditions. 

Superelastic NiTi wires usually had A_f_ below room temperature [2,7-8]. The transition between phases occurred under the effect of both temperature and stress within interposition in R-phase [8] and contains in its composition both nickel and titanium, and a little amount of an additional metal, which is usually added to improve the mechanical properties and reduce hysteresis and fraction between brackets and wires [2-6].

A_f_ of superplastic wires NT3-SE (Ni-Ti-Cr;50-48-2) that were used in the current study was 16.84°C, which is below room temperature. Therefore, these wires will transition from the R-phase to the austenite phase at room temperature; however, it also contains the chromium element in its composition. During thermal analysis, two peaks were detected that affect stress hysteresis and fatigue resistance.

Limitations

Two important limitations were encountered in the current study. The first is related to the employed archwires, whereas the second is related to the mechanical and thermal methods. For the first point, we used only rectangular wires (0.016 x 0.022 inch) manufactured by the same company, and therefore we could not agree with a lot of previous studies.

For the second point, the mechanical method (Instron 350M) that we used for the pending test was not designed to measure light forces, and therefore we had to adjust the load cell of the device to measure light force levels. That may have slightly affected our result. As for the thermal method (DSC), we settled for the heating turn due to device properties, and therefore we could not identify the transition temperature degree during cooling.

## Conclusions

Thermal wires showed lower forces than superelastic during the unloading stage, even though the differences were not statistically significant. The elemental composition was different between the superelastic wires and the thermal ones. This difference led to a difference in the produced force levels. The superelastic wires were also different from the other two types in terms of crystalline structure. Thermal wires with higher A_f_ temperatures showed lighter forces than those with lower A_f_ temperatures. The three types of archwires had an activation temperature degree located in the range of oral cavity variations. The transition in crystalline structure plays a major role in the mechanical behavior of NiTi wires.

## References

[1] Andreasen GF, Hilleman TB (1971). An evaluation of 55 cobalt substituted Nitinol wire for use in orthodontics. J Am Dent Assoc.

[2] Kusy RP (1997). A review of contemporary archwires: their properties and characteristics. Angle Orthod.

[3] Proffit WR, Fields Jr HW, Sarver DM (2006). Contemporary Orthodontics.

[4] Bellini H, Moyano J, Gil J, Puigdollers A (2016). Comparison of the superelasticity of different nickel-titanium orthodontic archwires and the loss of their properties by heat treatment. J Mater Sci Mater Med.

[5] Burstone CJ, Qin B, Morton JY (1985). Chinese NiTi wire--a new orthodontic alloy. Am J Orthod.

[6] Nespoli A, Villa E, Bergo L, Rizzacasa A, Passaretti F (2015). DSC and three-point bending test for the study of the thermo-mechanical history of NiTi and NiTi-based orthodontic archwires. J Therm Anal Calorim.

[7] Iijima M, Ohno H, Kawashima I, Endo K, Mizoguchi I (2002). Mechanical behavior at different temperatures and stresses for superelastic nickel-titanium orthodontic wires having different transformation temperatures. Dent Mater.

[8] Ohara AT (2016). Clinical importance of austenitic final point in the selection of nickel-titanium alloys for application in orthodontic-use arches. Revista odontológica mexicana.

[9] KAWASHIMA I, OHNO H, SACHDEVA R (1999). Relationship between Af temperature and load changes in Ni-Ti orthodontic wire under different thermomechanical conditions. Dent Mater J.

[10] Parvizi F, Rock W (2003). The load/deflection characteristics of thermally activated orthodontic archwires. The. Eur J Orthod.

[11] Higa RH, Henriques JFC, Janson G (2017). Force level of small diameter nickel-titanium orthodontic wires ligated with different methods. Prog Orthod.

[12] Lombardo L, Toni G, Stefanoni F, Mollica F, Guarneri MP, Siciliani G (2013). The effect of temperature on the mechanical behavior of nickel-titanium orthodontic initial archwires. Angle Orthod.

[13] Iijima M, Ohno H, Kawashima I, Endo K, Brantley W, Mizoguchi I (2002). Micro X-ray diffraction study of superelastic nickel-titanium orthodontic wires at different temperatures and stresses. Biomaterials.

[14] Bavikati VN, SINGArAjU GS, Mandava P, Killamsetty SS, Nettam V, Karnati PKR (2016). Evaluation of mechanical and physical properties of clinically used and recycled superelastic NiTi Wires. J Clin Diagn Res.

[15] (2022). ISO 15841:2014: Dentistry — Wires for use in orthodontics. https://www.iso.org/standard/62223.html.

[16] Berzins DW, Roberts HW (2010). Phase transformation changes in thermocycled nickel-titanium orthodontic wires. Dent Mater.

[17] Spini TS, Valarelli FP, Cancado RH, Freitas KMSd, Villarinho DJ (2014). Transition temperature range of thermally activated nickel-titanium archwires. J Appl Oral Sci.

[18] Thayer TA, Bagby MD, Moore RN, DeAngelis RJ (1995). X-ray diffraction of nitinol orthodontic arch wires. Am J Orthod Dentofacial Orthop.

[19] Kumar A, Konda P (2014). X-ray diffraction of orthodontic archwires for evaluation and comparison of surface deposits: an in vitro study. J Orthod Res.

[20] Iijima M, Brantley W, Guo W, Clark W, Yuasa T, Mizoguchi I (2008). X-ray diffraction study of low-temperature phase transformations in nickel-titanium orthodontic wires. Dent Mater.

